# Serum C-Reactive Protein Distribution in Minimally Invasive Total Knee Arthroplasty Do Not Differ with Distribution in Conventional Total Knee Arthroplasty

**DOI:** 10.1371/journal.pone.0124788

**Published:** 2015-04-24

**Authors:** Jean Cyr Yombi, Pierre Emmanuel Schwab, Emmanuel Thienpont

**Affiliations:** 1 Department of Internal Medicine and Perioperative Medicine, Cliniques Universitaires St Luc, Brussels, Belgium; Université Catholique de Louvain, Brussels, Belgium; 2 Department of Orthopaedics and Traumatology, Cliniques Universitaires St Luc, Brussels Belgium; Université Catholique de Louvain, Brussels, Belgium; Harvard Medical School, UNITED STATES

## Abstract

Minimally invasive total knee arthroplasty (MITKA) has been developed to reduce surgical trauma and facilitate rehabilitation after arthroplasty. A plausible hypothesis is that this reduced trauma results in lower concentrations of circulating inflammatory biomarkers, such as C-reactive protein (CRP). In this study, we compared CRP concentrations in patients undergoing MITKA to those undergoing conventional TKA (CTKA). Eight hundred and seven patients undergoing MITKA were prospectively recruited. CRP was measured before operation and on days 2, 4, 21, and 42 after operation. Two hundred and forty-seven patients who had CTKA were collected retrospectively, with the same inclusion and exclusion criteria as those who had MITKA. We found in both groups, that CRP values rose abruptly after operation, with peak values reached on day 2 or 4. Values then declined so that by days 21 and 42 they were only modestly above baseline values. Throughout the entire study period, mean CRP in MITKA patients did not differ significantly from those in CTKA patients. However, a significantly higher proportion of CTKA patients than of MITKA patients had peak CRP values at day 4 rather than at day 2 (76.8% vs 42.5%), a difference that was more pronounced in women. Also, by day 42, CRP values were still above baseline in 18.5% of MITKA patients and 28.8% of CTKA patients without known complications. In conclusion, CRP distribution pattern was similar in patients who received MITKA or CTKA,. CRP values remained slightly elevated in both MITKA and CTKA patients for as long 42 days after operation. These findings suggest that MITKA is no less traumatic than CTKA, as determined by CRP values, and the patterns of postoperative CRP may be useful in the management of TKA patients.

## Introduction

Total knee arthroplasty (TKA) is increasingly used to alleviate pain and improve mobility in osteoarthritic patients [1]. TKA is a major surgical stress and is associated with significant increases in postoperative circulating levels of plasma hormones and inflammatory markers [2]-[3]. The release of neurogenic substances from the surgical area into the innervated tissues contributes to the establishment of peripheral inflammation [2]. Local cytokines such as interleukin-6 and monocyte chemoattractant protein-1 have been linked to surgical trauma in knee arthroplasty [4]. Systemic inflammatory markers can be measured in the blood of surgical patients. Among those markers, C-reactive protein (CRP), an acute-phase protein produced by hepatocytes, is a commonly used biomarker; its plasma concentration increases during inflammatory states, a characteristic that has long been used for clinical purposes [5]. CRP serum concentrations in healthy adults are lower than 10 mg/L. CRP synthesis increases rapidly within hours of tissue injury or infection, which reflects the contribution of CRP to host defence and the innate immune response [5]. CRP concentrations rise after conventional knee arthroplasties (CTKA) and hip arthroplasties, thus reflecting systemically the trauma of the operations [6]-[7]. Some authors [6]-[13] have measured CRP concentrations after elective orthopaedic surgery and concluded that CRP reflects the extent of tissue damage during surgery, with peak values occurring at day 2.

Minimally invasive total knee arthroplasty (MITKA) has been developed to reduce trauma of the knee replacement and facilitate rehabilitation after the operation [14]. A plausible hypothesis is that this reduction in surgical trauma results in lower concentrations of circulating inflammatory biomarkers such as CRP. However, direct comparison of CRP in MITKA and CTKA in large series are scarce. [11]

In this study we have proposed that CRP concentrations will be.different between MITKA and CTKA. Specifically, the peak values at day 2.will. be lower after MITKA than after CTKA, and the CRP concentrations will return to normal sooner after MITKA than after CTKA.

## Materials and Methods

### Patients

The study was carried out in a 1,000-bed teaching hospital. We compared patients who had MITKA between January 1, 2010 and February 28, 2014 with those who had CTKA between November 30, 2000 and December, 23, 2005. Data were collected prospectively for MITKA and retrospectively for CTKA, using our institution’s operating software for patient medical records (Medical Explorer v3r9, Saint-Luc Hospital, 2008).

### CRP measurement

Serum CRP was measured by use of immunoturbidimetry on the Olympus AU2700, using Beckman Coulter reagents CRP Latex. The assay has a limit of detection of 0.07 mg/L with the High Sensitivity program and 0.14 mg/L with the normal program. The imprecision of the assay at the concentrations of 0.23, 9.7, 48.3, and 137 mg/L are 5.7, 1.9, 1.5 and 1.7%, respectively

### Exclusion criteria

Patients with clinical signs of infection, neoplasia, or inflammatory disease (rheumatoid arthritis, lupus erythematosus, or human immunodeficiency virus) and those who had undergone any operative procedure within the previous three months were excluded. Patients with CRP levels >10 mg/L before surgery and those who did not have three measurements of CRP during the study also were excluded.

### Surgical procedures

A single surgeon (ET) operated on each patient, performing MITKA using the medial parapatellar approach. Cephalosporins were given 1 h before tourniquet inflation. In the minimally invasive or limited medial parapatellar approach, the quadriceps tendon is incised for 2–3 cm, and the transcapsular incision is extended distally medial from the patella to the tibial tubercle. The suprapatellar pouch is no longer violated, the patella is no longer everted, and the tibia is not dislocated anteriorly. A posterior stabilised implant (Vanguard, Biomet, Warsaw, Poland) was used in all patients, with cemented components and patella resurfacing. Weight bearing was allowed the day after surgery, and range of motion exercises were started immediately after the operation. No drains were used. Nadroparin was administered for ten days after operation for prevention of thrombosis. For CTKA, a conventional parapatellar approach was used.

### Data collection

Patients’ demographics and clinical features were recorded (age, gender, weight, height, and body mass index [BMI]). For MITKA, blood samples were collected before operation (day 0) and on days 2, 4, 21, and 42 after operation as part of our standard arthroplasty follow-up. In order to detect early postoperative complications, all patients were examined every day during their five-day hospitalisation and at the outpatient clinic on days 21 and 42 after operation. All patients who met our inclusion criteria were followed for at least 6 weeks postoperatively.

For CTKA, data were collected retrospectively. We carefully reviewed the files of all patients and selected only those who met strict criterion of inclusion. These restrictions account for there being fewer patients in the CTKA group than in the MITKA group.

### Ethical issues

All patients were informed of the data collection and their future anonymous statistical analysis for MITKA. All patients agreed orally to take part in this study upon admission to the ward. Our institutional ethics committee stated that a written consent is not needed for analyses of anonymized data bases concerning data coming from routine practice, as permitted by country and European laws. Consequently, institutional ethical committee approval was granted for this study, and the committee approved this consent procedure. The surgeon ET explained the study to each patient on admission for MITKA. Before the blood sample was drawn, the study nurse discussed orally with the patient and asked permission to record the patient’s data in our file. For CTKA, data was retrospectively collected and the institutional ethical committee give its authorization (N°B403201 111 562 CEBH of the Université Catholique de Louvain, Brussels, Belgium).

### Statistical analysis

Statistical analyses were performed by use of Logiciel XLSTAT 2012. All values were expressed as mean±standard deviation (SD) when the distribution was normal, and expressed as symmetrical and median (min—max) when the distribution was right-skewed. After descriptive analyses, we did not perform further analyses, taking into account gender and BMI because the study included more female than male patients, and most patients had a high BMI. We performed a boostrap analysis by use of XLSTAT 2012 to allow robust determination. We performed a Student’s t test to compare CRP values at day 0, 2, 4, 21, and 42 of MITKA and CTKA patients.

## Results

In total, 807 patients who received MITKA and 249 who received CTKA met the inclusion criteria. Demographic data of the patients are summarized in Table 1. No significant differences between the groups in age or BMI were found, but the length of stay was significantly longer for the CTKA patients.

**Table 1:** Demographic and clinical features of MITKA and CTKA patients.

	MITKA	CTKA	P-value
Men (no.)	232	52	
Women (no.)	575	197	
Age (mean)	68.4	69.4	0.202
BMI^1^ (mean)	30	30	
Mean LOS^2^ (day)	4.41	13.6	0.000

^1^BMI, basal metabolic index;

^2^LOS, length of stay

**Fig 1:**
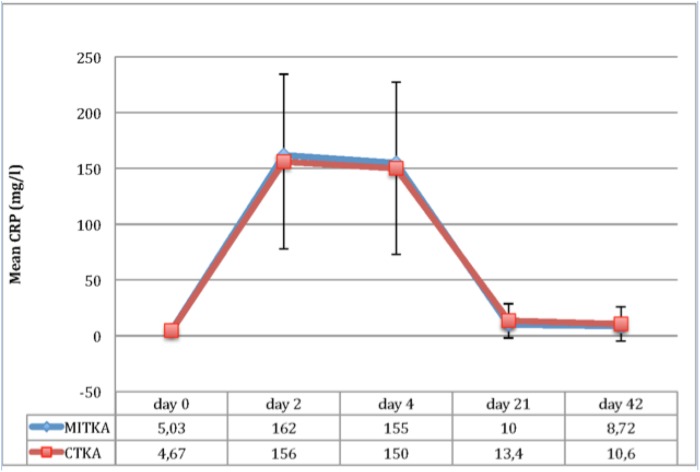
Evolution of CRP in the two TKA groups during six weeks(Values for the MITKA group compared to the CTKA group were not significantly different at any time point).

Fig 1 illustrate the mean CRP values at various days after the operations. In both the MITKA and CTKA groups, the mean CRP concentration rose markedly after operation, with peak levels reached on day 2. By day 21, values were only modestly above baseline values, and by day 42, 77.4% of MITKA patients and 71.2% of CTKA patients had normal CRP values. Values for the MITKA group compared to the CTKA group were not significantly different at any time point.

However, as illustrated in Table 2, peak CRP values were reached at postoperative day 4 (“delayed peak”) in significantly more CTKA patient than in MITKA patients. Also, peak values were reached at day 4 in significantly more women than men in both the MITKA and CTKA groups (p = 0.001).

**Table 2:** Percentage of patients with delayed peak of CRP at day 4 in the two groups.

	MITKA	CTKA*
Total	42.5	76.8
Men	32	18.6
Women	68	79.4

* P value = 0.000

## Discussion

In this study, we proposed that CRP concentrations would be.different after MITKA and CTKA, possibly due to less trauma and inflammatory reaction associated with MITKA. In fact, we found little differences in the CRP concentrations between the two surgical groups. Mean CRP concentratons were not significantly different at any time point (two, four, 21 and 42 days). We did find that significantly more CTKA patients had their peak CRP value at four days rather than two days. Whether this difference is of clinical significance is not known. Also, significantly more women than men had the four-day « delayed » peak CRP; the significance of this difference also is unknown.

Our study is one of the largest in which the chronological values of CRP after MITKA and CTKA have been compared. In our study, mean CRP levels on day 2 were 162 g/L and 156 mg/L for MITKA and CTKA, respectively, which is in the peak range recorded by others of 100 and 260 mg/L [6]- [7], [10]- [12]. Together, the most important finding of the studies is that CRP values after MITKA and CTKA are similar. Thus, it can be concluded that MITKA likely is not less traumatic than CTKA, at least as can be determined from systemic CRP measurements. This conclusion seems rational, as bone cuts in the two procedures are the same, and a possible difference would be observed only at the soft tissue level. In support of this possibility, we recently reported that the peak CRP level was higher in minimally invasive TKA and minimally Patient specific instrumentation(PSI)–assisted TKA than in unicompartmental arthroplasty. The difference between these three procedures is that less bone is resected in unicompartmental arthroplasty [15].

We did not observe a faster reduction in CRP levels in the MITKA group than in the CTKA group. Although at day 21 fewer patients reached normal CRP levels in the MITKA group than in the CTKA group, this difference was not statistically significant, a finding similar to that of published reports [16]. These results are clinically important because they highlight that although CRP usually returns to normal levels 3 to 6 weeks after operation [6], [8], [9], [11]- [12], [17]-[18], a significant number of patients (about one-fourth in our series) with uncomplicated TKA do not have normal CRP levels at that time. In accordance with this conclusion, Almeida Herrero *et al*. [18] reported that CRP values in uncomplicated conventional TKA cases decreased gradually to preoperative levels on Day 150 but were still high on Day 42.

Another potentially important finding is that in about 40% to 75% of our patients with uncomplicated MITKA or CTKA, CRP values did not peak until day 4, whereas reported maximum values have occurred between the 2^nd^ and 3^nd^ postoperative day [11]-[13], [17]. Our findings indicate that a slightly later peak in CRP concentrations, especially in women, may not be cause for concern.

The acute-phase response after surgery is diverse and complicated. Serum interleukin-6 levels, CRP levels, white blood cell counts, and fever are markers of surgical trauma. Some authors have studied CRP in various types of orthopaedic surgery [6]-[13], [19–23]. They concluded that CRP levels reflect the extent of tissue damage during surgery, and the increase in CRP depends not only on the amount of tissue injured but also on the type of tissue, such as bone, fat, or muscle, damaged [6]–[13], [15], [19–23]. Recently, Shen *et al*. [23] compared CRP concentrations after four types of arthroplasty and found that peak concentrations after hip resurfacing and computer navigation-assisted TKA were lower than those after conventional primary total hip arthroplasty and TKA. The last two procedures can be considered “systemic minimally invasive” arthroplasties [22]. Tsujii et al. [24] found no differences in CRP between a MITKA and a CTKA group, but did not specify the CRP values. Birgin *et al*.[16], in a prospective comparison of minimally invasive total hip arthroplasty procedures, found a 5.5-fold difference in levels of creatinine kinase, a marker for muscle damage, but no difference in the levels of general inflammatory cytokines (including CRP) between anterior and posterior approaches. The average increase in CRP levels after hip arthroplasty in the posterior group was slightly higher than in the anterior group, although no difference approached significance at any point in time or cumulatively. Niki et al. [25] studied the difference in surgical trauma of different minimally invasive approaches with tests of various muscle enzymes. They found no difference between conventional and minimally invasive surgery and even more muscle damage with a midvastus approach. Whether this measurement is a true measure of a procedure’s ‘‘invasiveness” is debated. Some authors argue that the similar results might be accounted for by the fact that the inflammatory cascade associated with hip arthroplasty is not greatly influenced by the surgical approach, and is defined rather by bone resection and implant placement [23]. CRP peak levels after TKA have been found higher than those after hip arthroplasy [6], [22]. TKA is more traumatic, and the severity of bone and bone marrow trauma seems to be an influencing factor, probably because macrophages are more numerous in bone than in muscle; after TKA, the degree of inflammation was higher and duration longer. In regard to this topic, it may be important that Moreschini et al. [26] found no differences in inflammatory markers between hip arthroplasty and TKA.

Several authors have tried to assess the value of maximum CRP levels and various established threshold levels, in diagnosing postoperative infection during arthroplasty. Dupont *et al*., [17] in their postoperative monitoring of lower limb arthroplasty, found that a CRP level threshold of 25 mg/L was not sufficiently reliable for early detection of postoperative infections (at the surgical site or elsewhere). However, the 25mg/L threshold has a 100% specificity and positive predictive values. A CRP threshold of 18 mg/L is not better, because even though it yields a slightly higher sensitivity (66.7%), it strongly decreases specificity (88.9%). Codine *et al*., [27] drawing a receiver operating characteristic curve, defined a CRP level of 12 times the normal range (regardless of the postoperative period) as a diagnostic element for surgical site infection, with a sensitivity of 100%, a specificity of 83.6%, a negative predictive value of 100%, and a positive predictive value of 25.9%. According to those authors, a CRP level of 60 mg/L favors puncture or surgical care, despite its low specificity. Our study did not confirm this assessment despite the fact that it was not designed for this purpose. Thus, the diagnosis of septic complications should be based on clinical findings rather than on CRP values [28].

Our study has limitations. One is that CRP was the only measurement of systemic inflammatory responses monitored, and it may not reflect activity of the entire inflammatory cascade. Another limitation is that the CTKA group was analysed retrospectively. Despite these limitations, we feel that our results are useful for clinicians delivering postoperative care of patients with TKA.

We conclude that 1) the marker of inflammatory reactions CRP is not substantially different in patients who have MITKA compared with those who have CTKA. Thus, MITKA evidently does not induce significantly less inflammatory reaction than does CTKA. 2) A slight delay in peak CRP values (from day 2 to day 4), especially in women, is common in TKA and likely is not a matter of concern. 3) A substantial portion of patients with uncomplicated TKA have slightly elevated CRP levels 3 to 6 weeks after the operation procedure, and this finding also may not indicate the presence of a postoperative complication, such as sepsis. 4) Serial measurement of CRP concentrations, rather than isolated measurements, may be more useful in the early detection of complications after TKA
